# MST1, a key player, in enhancing fast skeletal muscle atrophy

**DOI:** 10.1186/1741-7007-11-12

**Published:** 2013-02-01

**Authors:** Bin Wei, Wen Dui, Dong Liu, Yan Xing, Zengqiang Yuan, Guangju Ji

**Affiliations:** 1 National Laboratory of Biomacromolecules, Institute of Biophysics, Chinese Academy of Sciences, Datun Road 15, Beijing 100101, China; 2 State Key Laboratory of Brain and Cognitive sciences, Institute of Biophysics, Chinese Academy of Sciences, Datun Road 15, Beijing 100101, China

**Keywords:** MST1, muscle atrophy, FOXO3a

## Abstract

**Background:**

Skeletal muscle undergoes rapid atrophy upon denervation and the underlying mechanisms are complicated. FOXO3a has been implicated as a major mediator of muscle atrophy, but how its subcellular location and activity is controlled during the pathogenesis of muscle atrophy remains largely unknown. MST1 (Mammalian Sterile 20-like kinase 1) is identified as a central component of the Hippo signaling pathway. MST1 has been shown to mediate phosphorylation of FOXO3a at Ser207. Whether this MST1-FOXO signaling cascade exerts any functional consequence on cellular homeostasis remains to be investigated.

**Result:**

We identified that MST1 kinase was expressed widely in skeletal muscles and was dramatically up-regulated in fast- but not slow-dominant skeletal muscles immediately following denervation. The results of our histological and biochemical studies demonstrated that deletion of MST1 significantly attenuated denervation-induced skeletal muscle wasting and decreased expression of *Atrogin-1 *and *LC3 *genes in fast-dominant skeletal muscles from three- to five-month-old adult mice. Further studies indicated that MST1, but not MST2, remarkably increased FOXO3a phosphorylation level at Ser207 and promoted its nuclear translocation in atrophic fast-dominant muscles.

**Conclusions:**

We have established that MST1 kinase plays an important role in regulating denervation-induced skeletal muscle atrophy. During the early stage of muscle atrophy, the up-regulated MST1 kinase promoted progression of neurogenic atrophy in fast-dominant skeletal muscles through activation of FOXO3a transcription factors.

## Background

Skeletal muscle atrophy occurs upon denervation and disuse [1], as well as during aging and [2] under many catabolic disease states, including cardiac and renal failure, sepsis, cancer cachexia and diabetes [3-7]. During the pathogenesis of muscle atrophy, protein synthesis is significantly suppressed largely due to the inhibition of the IGF-1/PI3K/Akt pathway [8]. Protein degradation is accelerated due to activation of both the ubiquitin-proteasome system and autophagy, which work coordinately to remove the myofibrillar proteins and myocyte organelles and thus results in rapid loss of muscle mass and contractile function [9,10]. Expression of two E3 ligase genes, *Atrogin-1 *(*MAFbx*) and *MuRF-1*, as well as several autophagy genes, including *LC3, Bnip-3 *and *Gabarapl1*, are shown to be rapidly up-regulated at an early stage of multiple types of muscle atrophy [9,11,12]. FOXO3a transcription factor is able to bind to its consensus DNA-binding motif - TTGTTTAC - in the promoter regions of several of these atrogenes, including *Atrogin-1, LC3, Bnip3 *and *Gabarapl1 *[13-15]. Transactivation of these atrogenes by FOXO3a together with other transcription regulatory mechanisms, including NFκB and myogenic factors, results in the initiation of muscle atrophy [13,16-18].

The subcellular localization and activity of FOXO transcription factors are regulated by multiple post-translational modifications (PTM), including phosphorylation, ubiquitination and acetylation [19,20]. Some of these modifications of FOXO factors have been reported to regulate its activity and subcellular location in skeletal muscle [21-25]. Under physiological conditions, in response to insulin and/or IGF-1 signaling, FOXO transcription factors are phosphorylated and thus inhibited by the IGF-1/PI3K/Akt pathway. Akt kinases phosphorylate FOXO at multiple sites and create binding sites for 14-3-3 proteins, causing sequestration of FOXO factors in the cytosol [26]. In response to atrophy stimuli, activity of IGF-1/PI3K/Akt pathway is largely decreased [27], which results in the dephosphorylation of FOXO transcription factors, allowing its dissociation with 14-3-3 proteins and thus nuclear translocation [20,28]. Beyond phosphorylation and inhibition of FOXO by Akt kinases, whether there is any other regulatory mechanisms functioning upstream of FOXO factors during the pathogenic process of skeletal muscle atrophy remains to be investigated.

The mammalian Ste20-like kinase (MST) family is widely expressed in multiple tissues and consists of four members: MST1, MST2, MST3 and MST4 [29-34]. MST1 kinase was shown to be activated under multiple stress conditions, including oxidative stress [29] and activation of caspases [35,36]. As a central component of the mammalian Hippo signaling cascade [37], MST1 transcript was found in multiple skeletal muscles and in cardiac muscle [38]. In cardiac muscle, MST1 kinase has been shown to co-localize with and be capable of phosphorylating cardiac troponin I *in vivo *[39]. During the pathological process of myocardial infarction and hypertension-induced cardiac hypertrophy, MST1 kinase is activated and results in apoptosis of cardiomyocytes [40].

It has been identified that MST1 was activated through cleavage by caspase-3 during *in vitro *differentiation of the myoblast [41]. Cleaved MST1 translocates into the nucleus and promotes chromatin condensation and myotube differentiation. Whether MST1 plays any role during *in vivo *differentiation of skeletal muscle is unknown. Under *in vivo *conditions, fiber type composition of mammalian skeletal muscles is well determined during postnatal development [42,43]. Immediately after birth, multiple mouse skeletal muscles contain both slow- and fast-twitch fibers; following postnatal development and growth, fiber type composition of skeletal muscles is fully differentiated and maintained throughout adulthood before aging. However, the underlying molecular mechanisms are still largely elusive.

It has been identified that a conserved MST-FOXO signaling pathway regulates subcellular localization and transactivation function of FOXO transcription factors in neurons [30]. Upon oxidative stress, neuronal MST1 kinase is activated and phosphorylates FOXO proteins at a conserved serine site. The phosphorylated FOXO factors dissociate with 14-3-3 proteins and translocate into neuronal nucleus to activate its downstream pro-apoptotic genes, thus resulting in apoptosis of the neuron [30]. However, whether the function of this conserved MST-FOXO signaling pathway is fundamental in regulating any pathophysiological process *in vivo *is still not well understood.

In the present study, we examined expression of MST1 in multiple striated muscles during postnatal development. We observed that the protein level of MST1 was enriched at early postnatal stages and dramatically declined in adulthood. In order to elucidate whether MST1 plays any role during the pathological remodeling process of adult skeletal muscles, we performed sciatic denervation on hindlimb muscles from three- to five-month-old adult wild type (WT) and MST1 knockout (KO) mice. Through biochemical and histological studies, we identified that MST1 kinase was a key regulator of denervation-induced atrophy in fast- but not in slow-dominant skeletal muscles. Immediately following denervation, MST1 promoted increases in gene expression of *LC3 *and *Atrogin-1 *through activation of FOXO3a. MST1 deficient fast-dominant muscles in three- to five-month-old adult mice showed significantly attenuated progression of denervation-induced atrophy. Thus functioning upstream of FOXO3a transcription factor, MST1 kinase provides a potential therapeutic target for the clinical treatment of skeletal muscle atrophy.

## Results

### MST1/2 kinases are widely expressed in multiple skeletal muscles and enriched at the neonatal stage

**Figure 1:**
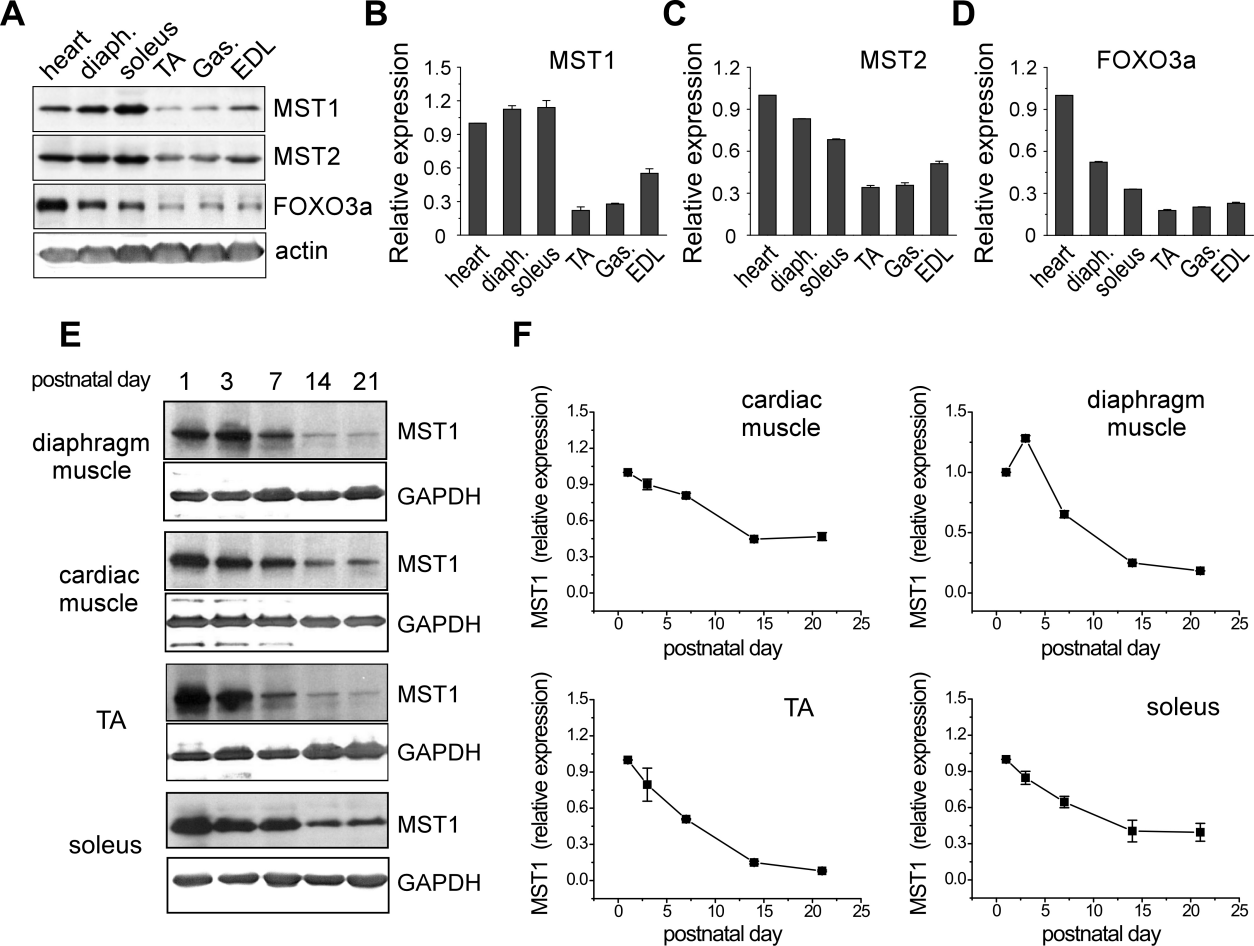
**MST1 is expressed in striated muscles and enriched at early stage of postnatal muscle development**. **A**. Expressions of MST1/2 and FOXO3a in striated muscles from three-month-old adult wild type mice were identified by Western blotting. **B-D**. Densitometry analysis showed the expressions of MST1/2 kinases were enriched in cardiac muscle, diaphragm as well as soleus muscles, which contain significant amount of type I fibers. **E**. Representative blotting shows protein level of MST1 was decreased during postnatal development in diaphragm, cardiac, TA and soleus muscles. **F**. Quantification of muscular MST1 expression during postnatal development.

In order to investigate the function of MST1 under pathophysiological conditions, we initially examined protein expression of MST1/2 kinases and their downstream transcriptional factor, FOXO3a, in multiple striated muscles, including cardiac muscle, diaphragm muscle, soleus (Sol), tibial anterior (TA), gastrocnemius (Gas) and extensor digitorum longus (EDL) muscles (Figure 1A). Densitometry analysis revealed that expression of MST1/2 kinases and FOXO3a was more predominant in cardiac muscle and slow fiber-dominant soleus muscles, compared with fast-dominant TA, gastrocnemius and EDL muscles (Figure 1B-D). Although it has been shown that transcripts of mammalian Hippo signaling components are found in multiple skeletal muscles and cardiac muscle [38], our data identified that the amount of protein of MST1/2 kinases was enriched in slow-dominant muscles and cardiac muscle. This suggests differential roles played by MST1/2 kinases in maintaining physiological homeostasis of different striated muscles.

To investigate whether the differential expression of MST1 kinase in fast- and slow-dominant muscles is established during postnatal development, we examined expression of MST1 kinase in fast- and slow-dominant muscles at different postnatal stages (Figure 1E, F). The results of Western blotting and densitometry revealed that the amount of MST1 kinase protein was at its highest level on postnatal Day 1 in cardiac muscle and multiple skeletal muscles, including the diaphragm, soleus and tibial anterior muscles (Figure 1E, F). From postnatal Day 1 to Day 21, protein levels of MST1 were continuously down-regulated in both slow-dominant soleus and fast-dominant TA muscles. At postnatal Day 21 (wean age), MST1 kinase protein level in soleus muscle remained 30.2 ± 0.01% of that at postnatal Day 1. In fast-dominant TA muscle at postnatal Day 21, MST1 protein level remained barely detectable. Thus, MST1 kinase decreased in soleus muscle at a slower rate compared with that in fast-dominant TA muscle, suggesting that differential expression of MST1 kinase in slow- versus fast-muscle is determined during postnatal development of skeletal muscles. Our data identified the differential expression pattern of MST1 kinase in slow- versus fast-dominant muscles and implicated the MST1 gene as potentially regulated by the postnatal myogenic regulatory program.

### MST1 kinase is up-regulated during muscle atrophy

In order to elucidate the function of MST1 during the pathological remodeling process of adult skeletal muscles, we performed sciatic denervation on hindlimb muscles from three- to five-month-old adult WT and MST1 KO mice. It has been shown in the literature that seven days after denervation, protein degradation mechanisms were fully activated within myofibers [9,11]; and fourteen days after denervation, muscle mass and myofiber size were both dramatically decreased compared with contralateral level [16]. We, therefore, collected contralateral and denervated muscle samples at denervation Days 3, 7 and 14, and examined transcription of muscle atrophy genes, and compared muscle mass and myofiber size.

**Figure 2:**
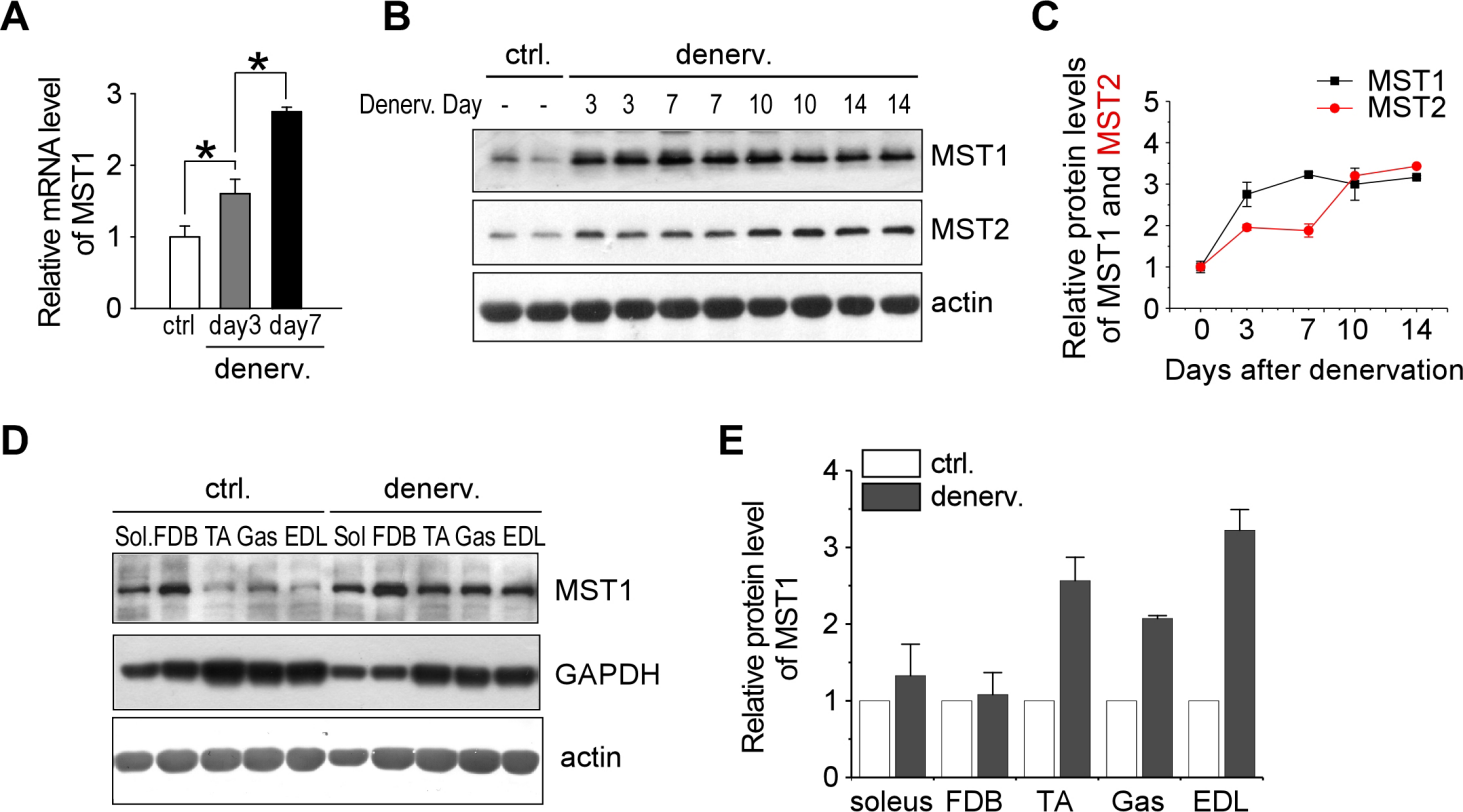
**MST1 kinase is dramatically increased during denervation-induced atrophy in fast-dominant muscles from adult mice**. **A**. Quantitative PCR showed mRNA of MST1 was continuously increased three and seven days after denervation. **B **and **C**. Western blotting and densitometry showed MST1 and MST2 protein levels were both increased during the process of denervation-induced atrophy in TA muscle. **D and E**. Seven days after denervation, MST1 protein levels were dramatically increased in multiple fast-dominant muscles (TA, Gas and EDL), but not in slow-dominant soleus and FDB muscles. *P < 0.05 by two-tailed Student's test.

Quantitative PCR revealed that MST1 gene transcription was up-regulated at three and seven days after denervation (Figure 2A). Western blotting also revealed that protein levels of MST1 and MST2 kinases were immediately up-regulated in TA muscle following denervation (Figure 2B, C). One week after denervation, the increase in MST1 kinase occurred in fast-dominant TA, Gas and EDL muscles, but much less dramatic in slow-dominant soleus and flexor digitorum brevis (FDB) muscles (Figure 2D, E), indicating MST1 kinase function regulates neurogenic muscle atrophy in a myofiber type-dependent manner. Thus, MST1 kinase function provides a potential mechanism mediating fast- but not slow-dominant muscle atrophy.

We also observed increased MST1 kinase protein level in gastrocnemius muscle following two weeks of hindlimb unloading (Additional file 1). Interestingly, MST2 protein level was not altered in this model of muscle atrophy, indicating MST1 and MST2 kinases play differential roles in mediating the occurrence of muscle atrophy. In a 48-hour starvation-induced muscle atrophy model, neither MST1 nor MST2 kinase protein level was altered in the gastrocnemius muscle examined (Additional file 1).

### MST1 deficient fast-dominant skeletal muscles are resistant to denervation-induced atrophy

The rapid increase in MST1 protein levels in fast-dominant muscles during denervation-induced atrophy suggests that MST1 kinase may functionally contribute to the muscle wasting process. However, it might also reflect an accumulation of undegraded protein in atrophying myofibers. In order to elucidate whether the up-regulated MST1 kinase exerts any functional influence on neurogenic muscle atrophy, we examined atrophy process in muscles from three- to five-month-old adult WT and MST1 KO littermate mice. MST1 KO mice were generated as previously described and was a kind gift from Dr. Wufan Tao [44]. Genomic deletion of exon 4 resulted in a reading frame shift and a premature stop codon in exon 5 [44]. We initially observed the normalized muscle mass of fast-dominant TA and gastrocnemius muscles were decreased in adult MST1 KO mice (Additional file 2). It has been demonstrated that MST1 was activated during myoblast differentiation [41]. However, whether MST1 is necessary for myogenic differentiation of myoblast *in vivo *is unknown. Our data suggest that MST1 expression was predominant in neonatal mouse skeletal muscles; MST1 was potentially involved in regulating postnatal development of fast-dominant muscles, and represents a neonatal gene program in skeletal myofiber.

**Figure 3:**
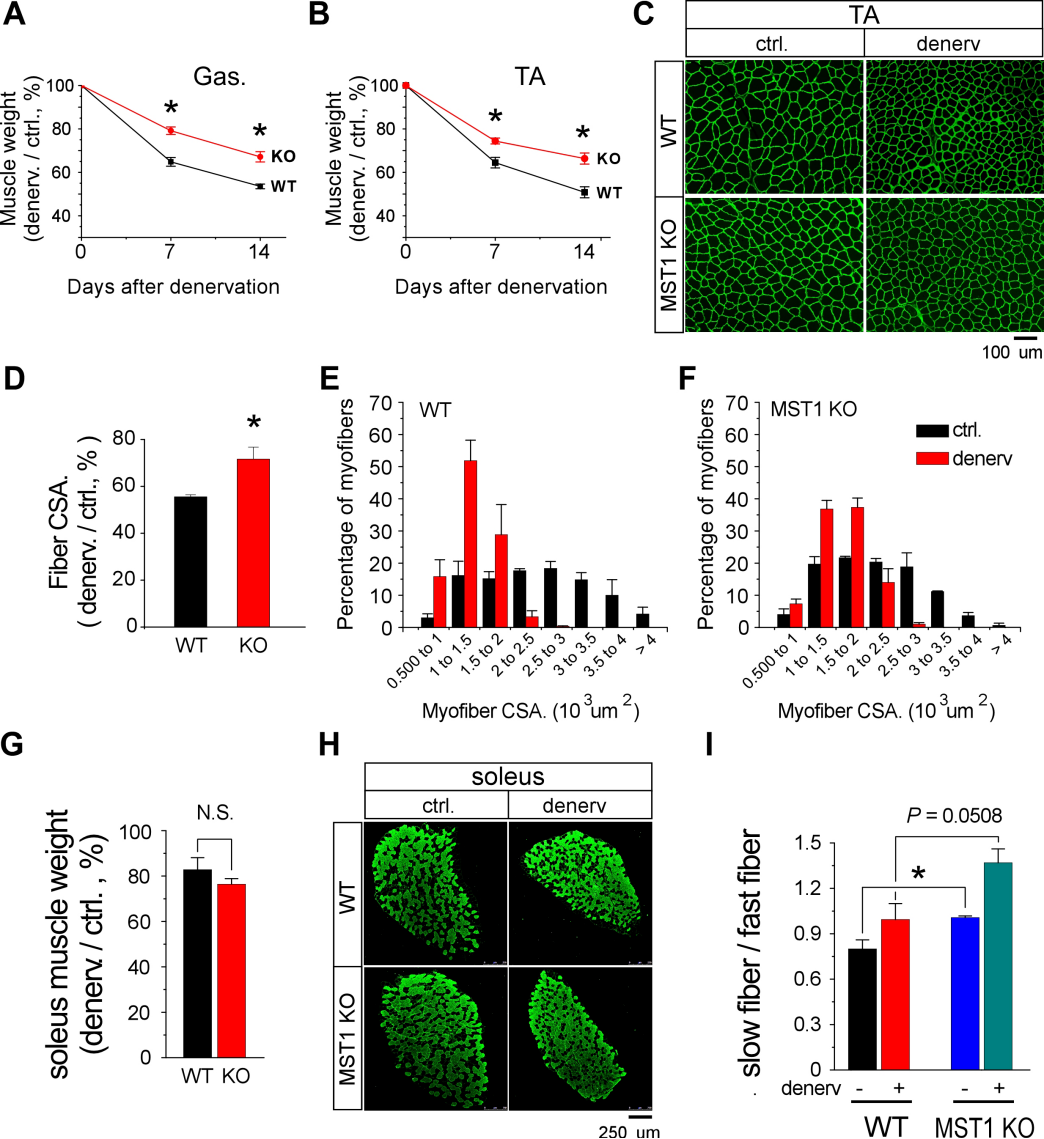
**MST1 deficiency protects fast- but not slow-dominant skeletal muscles against denervation-induced atrophy**. Sciatic denervation was performed on three- to five-month-old adult WT and MST1 KO mice littermates. Three pairs of WT and MST1 KO mice were sacrificed at Day 7 after denervation and the other three pairs were examined at Dday 14. **A**. Mass of denervated gastrocnemius muscles from MST1 deficient mice remained at 79.18% ± 1.73% (on Day 7) and 67.14% ± 2.37% (on Day 14) of the contralateral muscles, both of which were significantly higher than 64.83% ± 2.01% and 53.53% ± 1.09% in WT mice, respectively. **B**. Seven and 14 days after denervation, mass of denervated TA muscles from MST1 KO mice remained at 74.43% ± 1.36% and 66.35% ± 2.56% of contralateral levels, whereas WT TA muscle mass remained at 64.47% ± 2.4% and 50.81% ± 2.5%, respectively. **C**. Representative images of α-laminin staining on contralateral and denervated TA muscle cryosections, indicating myofiber size was better preserved in MST1 deficient TA muscles at Day 14 after denervation. **D**. Fourteen days after denervation, averaged myofiber cross-sectional area of denervated TA muscle decreased to 71.61% ± 5.11% of the contralateral level in MST1 KO mice and 55.56% ± 0.87% in WT mice. **E and F**. TA myofiber size distribution showed a suppressed leftward shift in MST1 deficient TA muscles (F) 14 days after denervation, indicating less atrophy compared with WT muscles (E). **G**. Fourteen days after denervation, WT soleus muscle mass remained at 82.78% ± 5.32% of contralateral and MST1 deficient soleus muscle mass remained at 76.43% ± 2.45% of its contralateral level. **H**. Representative images of MHC I staining on contralateral and post-denervated Day 14 soleus muscles from WT and MST1 KO mice. WT and MST1 deficient soleus muscles developed similar level of atrophy 14 days after denervation. **I**. The ratio of slow-to-fast fiber content was significantly higher in MST1 deficient contralateral soleus muscles than that of WT muscles. 14 days after denervation, the ratios of slow-to-fast fiber content in WT and MST1 deficient soleus muscle were both increased. * *P *< 0.05 compared with wild type by two-tailed Student's test.

We examined the pathological process of denervation-induced atrophy in adult WT and MST1 KO mice, by measuring muscle mass and myofiber size. Muscle mass data revealed that MST1 deficient gastrocnemius (Gas) and TA muscles were significantly resistant to denervation-induced muscle wasting (Figure 3A, B). Seven and fourteen days after denervation, wild type TA muscle mass decreased to 64.47% ± 2.4% and 50.81% ± 2.5% of contralateral level, respectively; while MST1 deficient TA muscle mass remained at 74.43% ± 1.36% and 66.35% ± 2.56% of its contralateral level (Figure 3B). Gastrocnemius muscle mass data measured seven and fourteen days after denervation also indicated that MST1 deficiency protected the muscle against denervation-induced atrophy (Figure 3A). Myofiber area quantification using alpha-laminin staining on TA muscle cryosections also revealed better preservation of myofiber size in MST1 deficient TA muscles fourteen days after denervation (Figure 3C). Although the myofiber size of MST1 deficient TA muscles was generally smaller than that in WT muscles under innervated conditions, normalized myofiber area in denervated MST1 deficient TA muscle was better preserved by more than 15% than that of WT myofibers on average (Figure 3D). Fourteen days after denervation, myofiber size distribution calculated from MST1 deficient TA muscles showed a delayed leftward shift from its contralateral conditions (Figure 3E, F). Thus, we identified that deficiency in MST1 kinase protected fast-dominant skeletal muscles against denervation-induced atrophy. The rapid increase in MST1 immediately following denervation potentially represents a pro-atrophy mechanism.

### MST1 deficiency does not prevent denervation-induced atrophy in slow-dominant muscle

Next we sought to examine whether MST1 kinase deficiency also affects the progression of denervation-induced atrophy in slow-dominant soleus muscles from adult mice. Western blotting and densitometry revealed that, unlike in fast-dominant muscles, protein levels of MST1 and MST2 kinases were not altered in atrophic soleus muscles compared with contralateral levels (Additional file 3). Two weeks after denervation, WT soleus muscle mass decreased to 82.8% ± 0.05% and MST1 deficient soleus muscle mass remained 76.4% ± 0.03% of their contralateral levels (Figure 3G), indicating that MST1 deficiency did not prevent the progression of denervation-induced atrophy in slow-dominant soleus muscle.

The enriched expression of MST1 kinase in slow-dominant soleus muscle encouraged us to investigate whether MST1 kinase activity contributes to the determination of muscle fiber type composition within the skeletal muscles. In diaphragm muscle obtained from adult MST1 deficient mice, type I fiber content was not significantly different from that of WT littermates (Additional file 4), indicating that MST1 kinase deficiency did not affect diaphragmatic myofiber composition. Interestingly, we observed a mildly increased content of type I fiber (Figure 3H, I) in MST1 deficient soleus muscle. In soleus muscles from adult wild type mice, type I fibers account for about 44% of total muscle content; while in MST1 deficient soleus muscles, about 50% of fibers are MHC I expressing type I fibers, suggesting that MST1 kinase deletion leads to a moderate slow-ward fiber type transition of soleus muscle (Figure 3I).

### MST1 deficiency reduces FOXO3a-dependent transcription of *Atrogin-1 *and *LC3 *genes in muscle atrophy

**Figure 4:**
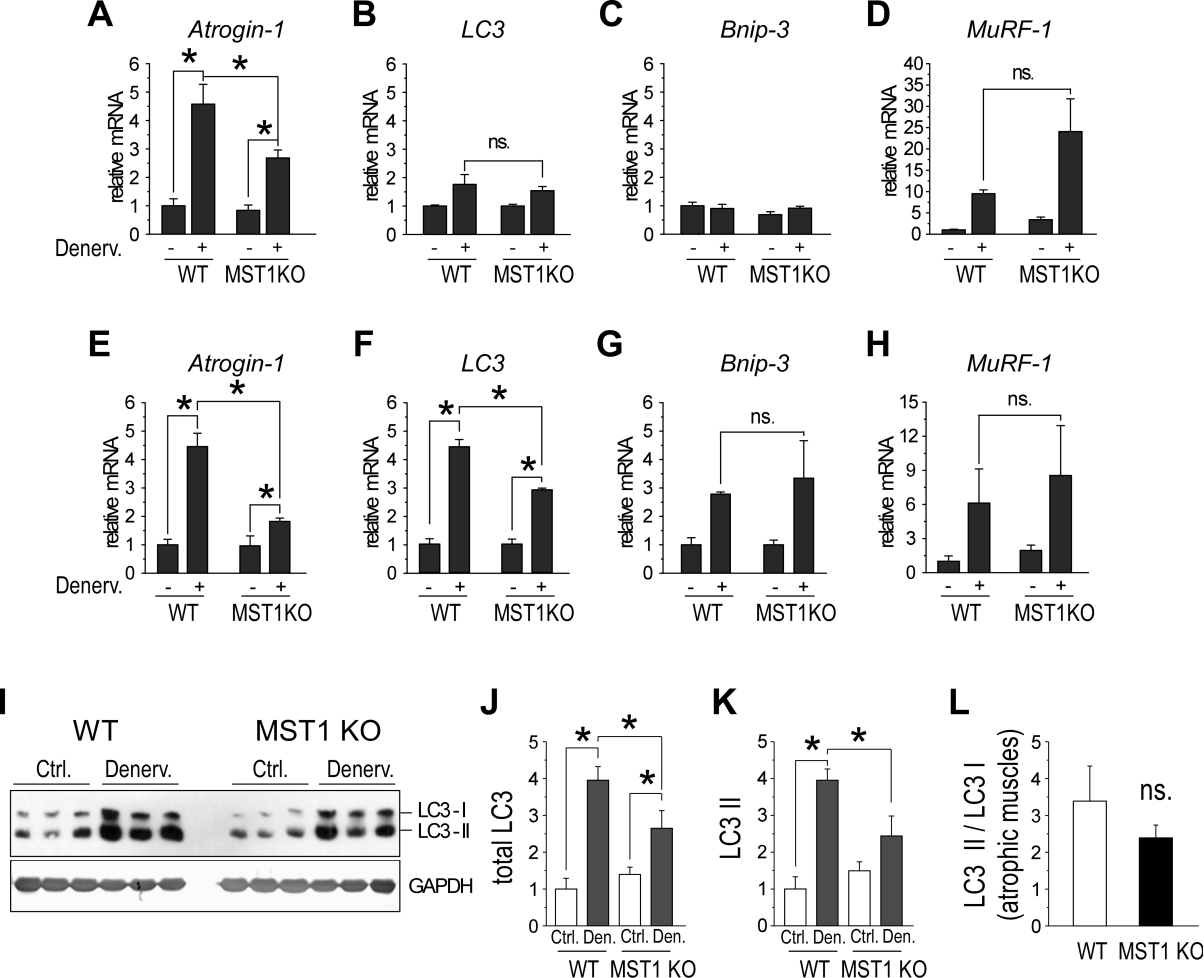
**Expression of atrophy related genes in denervated gastrocnemius muscles from WT and MST1 KO mice**. **A-D**. Quantitative PCR results showed three days after denervation, *Atrogin-1 *mRNA levels in WT and MST1 deficient gastrocnemius muscle were both increased (by 4.57 ± 0.70 folds and 2.68 ± 0.28 folds of the contralateral level, respectively). The increases in mRNA levels of *LC-3 *(1.76 ± 0.35 and 1.54 ± 0.15, WT vs. MST1 KO) and Bnip-3 were both less than two-fold and there is no significant difference in the WT and MST1 deficient muscles. *MuRF-1 *mRNA levels were upregulated to 9.51 ± 0.88 and 24.07 ± 7.68 folds in WT and MST1 deficient muscles, respectively. **E-H**. At Day 7 after denervation, the mRNA levels of muscle atrophy-related genes were significantly increased in both WT and MST1 deficient denervated muscles. The mRNA levels of *Atrogin-1 *and *LC3 *in denervated WT muscles (4.45 ± 0.47 and 4.43 ± 0.26 folds increased, respectively) were both significantly higher than that of MST1 KO mice (1.82 ± 0.12 folds and 2.91 ± 0.06 folds, respectively). *Bnip-3 *and *MuRF-1 *mRNA levels were also increased in denervated WT and MST1 deficient muscles but showed no differences between the genotypes. **I-L**. The protein levels of total LC3 and lapidated form (LC3 II) were both increased in MST1 deficient and WT muscles. Total LC3 protein was increased at 3.95 ± 0.37 folds in WT denervated muscle and 2.65 ± 0.48 folds in MST1 deficient denervated muscles. The LC3 II protein level in WT denervated muscle was increased 3.95 ± 0.31 folds and 2.44 ± 0.54 folds in MST1 deficient muscles, respectively. The ratio of LC3 II/LC3 I was 1.68 ± 0.20 and 1.31 ± 0.12 in WT and MST1 deficient muscles after denervation. * *P *< 0.05 by two-tailed Student's test.

The atrophy resistant phenotype of MST1 deficient fast muscles encouraged us to investigate the expression of the genes functionally involved in protein degradation and autophagy during muscle atrophy, including the two major E3 ligases identified in muscle wasting studies, *Atrogin-1 *and *MuRF-1*, as well as *LC3 *and *Bnip3*, which are involved in an activated autophagy pathway [9,45]. Through quantitative PCR, we observed significant increases in expression of *Atrogin-1 *and *MuRF-1 *genes in both WT and MST1 deficient atrophic gastrocnemius muscles at post-denervation Day 3 (Figure 4A, D). Expression of autophagy related *Bnip3 *and *LC3 *genes did not show apparent increases upon contralateral levels (Figure 4B, C), indicating activation of the ubiquitin-proteasome system is prior to activation of the autophagy pathway in early stages of neurogenic muscle atrophy. Thus, the degradation of myofiber proteins immediately following onset of atrophy is potentially through the ubiquitin-proteasome system. Interestingly, increase in *Atrogin-1 *gene expression in MST1 deficient gastrocnemius muscles was suppressed by approximately 40% compared with WT atrophic muscles (Figure 4A). Relative expressional levels of the *MuRF-1 *gene were higher in atrophic MST1 deficient muscles than that of WT although statistically not significant (Figure 4D). Seven days after denervation, the relative expressional levels of *Atrogin-1, MuRF-1, LC3 *and *Bnip3 *genes were all increased upon contralateral level in both WT and MST1 deficient atrophic muscles (Figure 4E-H). However, increases in expression of *Atrogin-1 *and *LC3 *were statistically less dramatic in atrophic MST1 deficient muscles compared with WT (Figure 4E, F), indicating that these genes were much less activated in MST1 deficient muscles compared with those of WT during muscle atrophy.

During the process of autophagy, LC3 I is converted to LC3 II through proteolysis and lipidation [46,47]. However, recent evidence showed that up-regulated autophagy activity was also accompanied by an increased total protein level of LC3 II but not the conversion ratio of LC3 II/I [48]. In our study, LC3 blotting and densitometry revealed up-regulation of total and activated LC3 proteins were less dramatic in MST1 deficient atrophic muscles than those of WT (Figure 4I-K). Although the ratio of conversion of LC3 into the activated forms (LC3 II/I) did not show a difference between WT and MST1 KO groups (Figure 4L), our data revealed that activation of *LC3 *gene expression in atrophic muscles was at least partially suppressed due to MST1 deficiency.

Among the genes examined in atrophic muscles, *Atrogin-1, Bnip3 *and *LC3 *were shown to be regulated by the FOXO3a transcription factor. FOXO3a is able to bind to the promoter regions of the genes encoding *Atrogin-1, LC3, Bnip3 *and *Gabarap1*, and induces dramatic increases in the expression of these atrogenes in muscles to initiate atrophy [13-15]. Thus, our results revealed that deficiency in MST1 kinase suppressed the activation of FOXO3a regulated genes in atrophic muscles, indicating that the activity of this transcription factor was differentially modulated in WT and MST1 deficient atrophic muscles.

### MST1 phosphorylates FOXO3a at Serine207 and promotes its nuclear translocation at early stage of muscle atrophy

It has been shown that FOXO3a shuffles into myofiber nucleus and its activity is up-regulated upon atrophy stimuli [13]. The subcellular location and activity of FOXO3a are precisely determined by multiple post-translational modification mechanisms [19]. The suppressed up-regulation of FOXO3a-mediated atrogenes in MST1 deficient fast muscles undergoing atrophy leads us to investigate whether the activation of FOXO3a is differentially modulated in atrophic WT and MST1 deficient muscles.

It has been previously reported that MST kinases are functionally upstream of FOXO transcription factors in a MST-FOXO signaling cascade. Through phosphorylating FOXO factors at a conserved serine site, MST kinases promote nuclear translocation of FOXO factors and activation of pro-apoptotic genes, thus promoting apoptosis of neurons upon oxidative stress [30]. In order to elucidate whether such a MST-FOXO signaling mechanism contributes to modulating the subcellular localization and activity of FOXO3a during the pathogenic process of neurogenic muscle atrophy, we examined the phosphorylation status of FOXO3a at Ser207 in both WT and MST1 deficient TA muscles undergoing atrophy.

**Figure 5:**
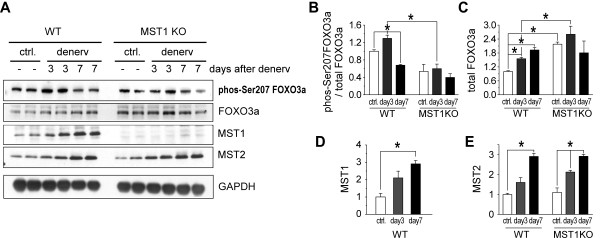
**MST1 phosphorylates FOXO3a at Ser207 during muscle atrophy**. **A**. Representative western blotting of MST1/2 and FOXO3a in atrophic TA muscles. **B**. Phosphorylation of FOXO3a at serine 207 by MST1 kinase was increased (by 29.5% ± 7.40%) in WT TA muscles but not in MST1 deficient muscles at denervation Day 3. FOXO3a Ser207 phosphorylation was decreased both in WT and MST1 KO muscles at Day 7 after denervation. **C**. Total FOXO3a protein level was continuously increased at Day 3 and Day 7 after denervation in WT TA muscles. **D **and **E**. Both the expression levels of MST1 and MST2 kinases were increased in atrophic muscles. MST2 protein levels were similarly increased in wild type and MST1 deficient muscles and WT muscles after denervation. At Dday 7 after denervation, comparing with the contralateral, the protein levels of MST2 kinase were increased 2.91 ± 0.15 and 2.92 ± 0.10 folds in the atrophic TA muscles obtained from WT and MST1 KO mice, respectively. * *P *< 0.05 by two-tailed Student's test.

We first confirmed the absence of MST1 protein in fast- and slow-dominant muscles from adult MST1 KO mice (Figure 5A and Additional file 3A). Western blotting and densitometry revealed three days after denervation, the phosphorylation level of FOXO3a by MST kinase at Ser207 was significantly up-regulated by 29.5% ± 0.07% (Figure 5A, B) in WT TA muscle, indicating the activation of FOXO3a transcription factor due to the up-regulation of MST kinases. Interestingly, the phosphorylation of FOXO3a at Ser207 was not increased in MST1 deficient muscles during atrophy, indicating that immediately following denervation, the phosphorylation of FOXO3a at Ser207 was mediated by MST1 but not MST2. Three and seven days after denervation, the FOXO3a protein amount was continuously increased in WT TA muscles and the total protein level of FOXO3a was consistently higher in MST1 deficient muscles (Figure 5C), suggesting a potential compensatory effect by FOXO3a gene expression under a MST1 deficient background. Three and seven days after denervation, the protein level of MST1 kinase was up-regulated in WT muscle (Figure 5A, D); while the protein level of MST2 kinase was also increased in both WT and MST1 deficient muscles to similar levels, indicating there was no functional compensation by MST2 kinase due to MST1 deficiency during muscle atrophy (Figure 5A, E). Further study indicated that seven days after denervation, the phosphorylation of FOXO3a by MST1 kinase was decreased in atrophic muscles. Thus the activation of FOXO3a was potentially tightly controlled by a dephosphorylation mechanism to restrict activation of this transcription factor.

**Figure 6:**
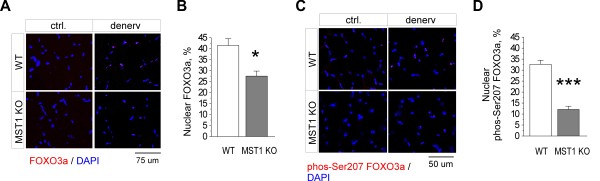
**MST1 promotes nuclear translocation of FOXO3a through phosphorylation at Ser207**. **A **and **C**. Representative immunostaining using pan-FOXO3a and Ser207-phosphorylated FOXO3a antibodies on TA muscle sections obtained on denervation Day 3. **B**. At Day 3 after denervation, 41.46% ± 3.07% and 27.51% ± 2.28% of the total myofiber nucleus were stained with FOXO3a in WT and MST1 deficient TA muscles, respectively. **D**. At Day 3 after denervation, 32.63% ± 1.82% and 12.10% ± 1.49% of total TA muscle nuclei stained with serine 207-posphorylated FoxO3a in WT and MST1 mice, respectively. *** *P *< 0.0001 by two-tailed Student's test..

It has been previously shown that phosphorylation of FOXO by MST kinases was sufficient to promote its dissociation from 14-3-3 proteins in cytosol and to promote nuclear accumulation of FOXO transcription factors [30]. In order to clarify whether MST1-mediated phosphorylation of FOXO3a also contributes to modulating its subcellular location during skeletal muscle atrophy, we examined the subcellular distribution of FOXO3a in atrophic muscles obtained from denervated adult MST1 deficient mice and their WT littermates. We observed a dramatic increase in nuclear localization of FOXO3a in both WT and MST1 deficient atrophic muscles (Figure 6A) at Day 3 after denervation. However, the level of nuclear accumulated FOXO3a in MST1 deficient muscles was significantly less compared to that in WT muscles (Figure 6B). In order to further elucidate whether the phosphorylation of FOXO3a on Ser207 is directly involved in its nuclear transportation, we examined the subcellular distribution of Ser207-phosphorylated FOXO3a on atrophic muscle sections. As shown in Figure 6C, D, nuclear localized Ser207-phosphorylated FOXO3a was significantly increased in WT atrophic muscles at Day 3 after denervation. In contrast, nuclear accumulation of Ser207-phosphorylated FOXO3a was dramatically suppressed in MST1 deficient muscles (Figure 6C, D).

Taken together, our results revealed that the up-regulated MST1 kinase was functionally involved in activation of FOXO3a through phosphorylation at Ser207, and thus promoted its nuclear translocation in fast-twitch myofibers undergoing atrophy; MST1 deficiency result in less activation of FOXO3a and suppressed nuclear translocation of this transcription factor, and thus lead to an atrophy-resistant phenotype in fast-dominant muscles from adult MST1 KO mice.

## Discussion

**Figure 7:**
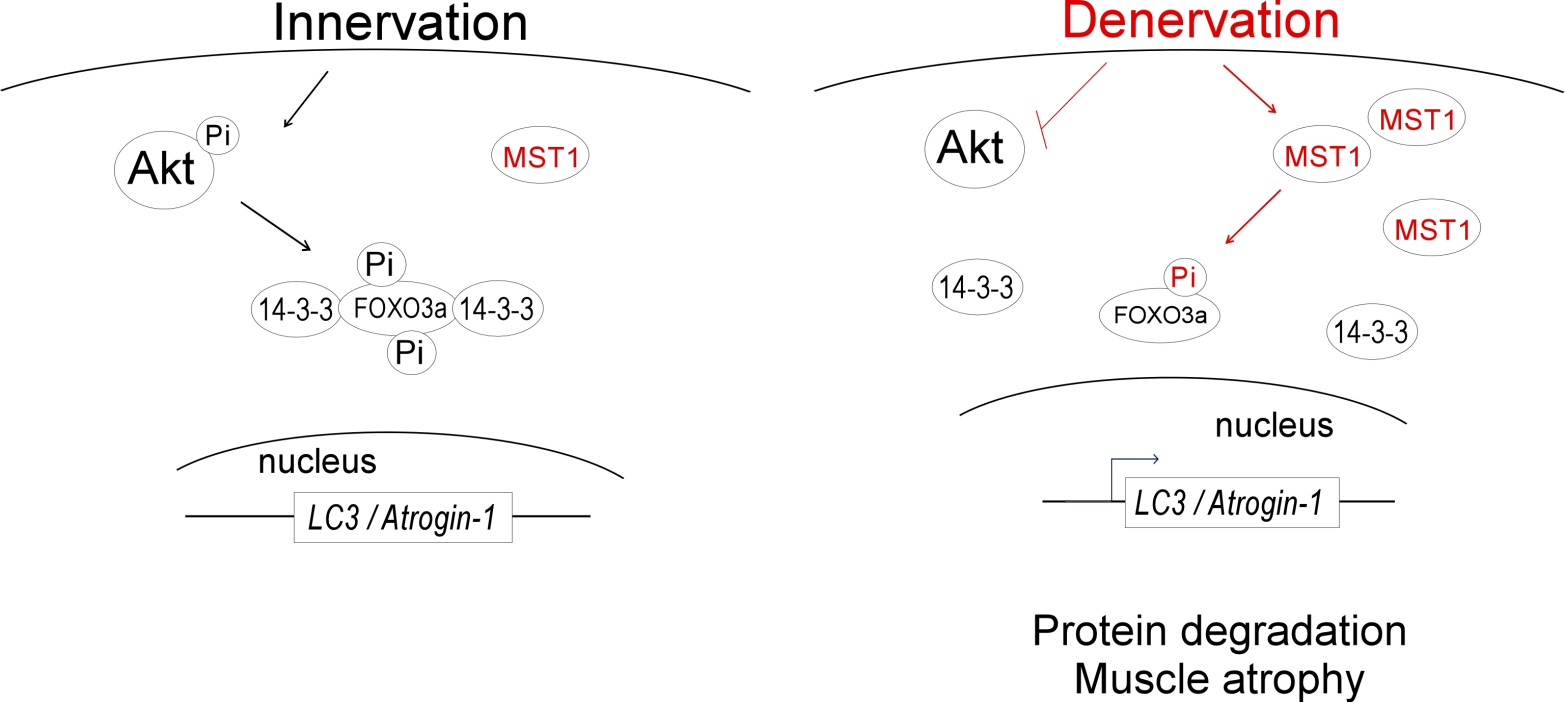
**Schematic diagram of MST1's function in mediating fast skeletal muscle atrophy**. Within an innervated myofiber, Akt kinase is active and MST1 kinase amount is restricted at low level. Thus FOXO3a is phosphorylated by Akt at multiple sites (Thr32, Ser253 and Ser315) [53], and is restricted within the myofiber cytosol through interaction with 14-3-3 proteins. Denervation induces rapid up-regulation of MST1 kinase. MST1 phosphorylates FOXO3a at Ser207 and causes its translocation into myofiber nucleus to activate expression of LC3 and Atrogin-1 genes, which leads to rapid muscle mass loss during the progression of muscle atrophy.

In the present study we have identified the role of MST1 kinase in mediating the pathologic process of neurogenic muscle atrophy. Upon denervation, MST1 kinase was immediately up-regulated in fast-dominant muscles. The up-regulated MST1 kinase directly phosphorylated FOXO3a transcription factor at Ser207, and promoted its nuclear translocation and activation of atrogenes (Figure 7).

MST1/2 was widely expressed in multiple striated muscles, including cardiac muscle, and slow- and fast-dominant skeletal muscles (Figure 1A). Interestingly, the expression of MST1 kinase was enriched in skeletal muscles at neonatal stages (Figure 1E, F), suggesting that MST1 kinase was potentially involved in regulating postnatal development of myofibers. It has been generally identified that a group of gene regulatory mechanisms functioning during early development of an organism are re-deployed under certain pathogenic conditions in adulthood. Here we identified that the MST1 gene expression was rapidly increased during the process of neurogenic fast muscle atrophy, thus MST1 represented one such re-deployed developmental gene program in fast muscles undergoing atrophy. Further studies using the MST1 deficient mouse model identified that the role of the up-regulated MST1 kinase was to promote the development of muscle atrophy, rather than functions as an adaptive mechanism to prevent rapid loss of muscle mass. We had also observed an enriched MST1 protein amount in slow-dominant soleus muscle compared with fast-dominant muscles under physiological conditions (Figure 1A, B). However, deficiency in MST1 leads to only a moderate switch in fiber type composition in soleus muscle (Figure 3 H). Thus, aims of future studies will be directed to investigate the role of MST1 in muscle development at early postnatal stages as well as whether MST1 contributes to maintaining the fiber type composition of skeletal muscle. In skeletal muscles, under both physiological and pathogenic conditions, MST1 kinase deficiency did not lead to expressional nor functional compensations by MST2 kinase (Figure 5A, E), suggesting MST1 and MST2 kinases play differential roles in maintaining the pathophysiological homeostasis of skeletal muscles.

It is well characterized that remodeling of gene expression in atrophic myofibers is orchestrated by multiple regulatory mechanisms [49]. FOXO3a transcription factor has been shown to mediate up-regulation of genes involved in both ubiquitin-proteasome and autophagy pathways [14]. Studies have shown that inhibition of FOXO3a in muscles treated with atrophic stimuli is capable of preventing muscle from atrophying [17,50]. We have previously shown that a conserved MST-FOXO signaling mechanism mediates the apoptotic response of neurons under oxidative stress [30]. In the present study, we have identified that the MST-FOXO signaling mechanism is also conserved in mouse skeletal muscles. MST1 phosphorylates and thus activates FOXO3a during neurogenic skeletal muscle atrophy, which provides a new molecular mechanism to precisely determine the subcellular location and activity of FOXO3a in myofibers undergoing atrophy.

Immediately following denervation, phosphorylation of FOXO3a at Ser207 was significantly enhanced (Figure 5A, B). Comparison between WT and MST1 deficient skeletal muscles further revealed that the phosphorylation of FOXO3a at Ser207 was mainly mediated by MST1, since a normalized level of Ser207-phosphorylated FOXO3a was consistently lower in MST1 deficient muscles under both normal and atrophic conditions. We had also observed an increased FOXO3a protein level in MST1 deficient fast muscles, which might reflect a functional compensation by FOXO3a for MST1 deficiency.

Our results revealed that the subcellular location of FOXO3a transcription factor in myofibers was controlled by both Akt kinase and MST1 kinase in a counter-balanced manner. Up-regulation of MST1 kinase function following denervation further promoted nuclear accumulation of FOXO3a and thus full development of muscle atrophy. FOXO3a transcription factor is able to bind to promoter regions of multiple atrophy related genes, including *Atrogin-1, LC3, Bnip*3, *Gabarapl1*. Among these atrogenes, we found that activation of FOXO3a by MST1 kinase especially affected the expression of *Atrogin-1 *and *LC3 *genes, but not *Bnip3*. Our results suggested that a differential post-translational modification status of FOXO3a might potentially result in activation of different groups of its target genes. However, the underlying mechanisms still need to be elucidated in further studies.

The transcriptional mechanism mediating *MST1 *gene expression is still not well understood, especially under multiple pathophysiological conditions. During muscle atrophy, many gene regulatory mechanisms have been reported to be activated to promote expression of protein degradation machineries, including myogenic factor myogenin and MyoD [16], NF-κB [18], as well as FOXO transcription factors [13]. The dramatic decrease in MST1 protein level in multiple striated muscles during postnatal development indicates that MST1 is potentially regulated by the muscle differentiation program. However, the molecular mechanisms mediating the expression of MST1 during both neonatal development and atrophic remodeling need to be determined in further studies. We have observed that both MST1 and MST2 kinase were up-regulated during fast-dominant muscle atrophy. However, MST2 could not compensate for MST1 deficiency in the aspects of phosphorylating FOXO3a and promoting myofiber atrophy. Thus, the molecular mechanisms differentiating the function of MST1 and MST2, as well as the function of MST2 during muscle atrophy, need to be elucidated in future studies.

The present study has focused on the biological consequence of an up-regulated MST1 kinase in atrophic muscles and its downstream molecular mechanisms. By elucidating the MST1-FOXO3a signaling mechanism in neurogenic muscle atrophy, we provide a potential therapeutic target for the clinical treatment of muscle atrophy.

## Methods

### Animal and muscle atrophy models

The use of animals and experimental procedures were approved by the Institutional Animal Use and Care Committee of Institute of Biophysics at Chinese Academy of Sciences. Generation of MST1 deficient mice was described previously [44]. For denervation studies, three- to five-month-old WT (n = 6) and MST1 KO (n = 7) mice, both male and female, were used. For denervation-induced muscle atrophy, mice were anesthetized, the sciatic nerve of left hind limb was exposed by blunt dissection and a 3 mm piece was excised. An identical incision into the right hind limb was made but the muscles remained innervated to serve as control. Muscle samples were harvested at 3, 7, 10 and 14 days after the denervation surgery. For disuse-induced muscle atrophy models, mice tails were suspended and fixed onto a mobile hook mounted to the cage. Hind feet of the mice were maintained 1 to 2 mm above the ground of the cage for 7 and 14 days. Mice were allowed free access to food and water during the suspension period. For starvation experiments, mice were deprived of food but allowed free access to water for 48 hours. Muscle atrophy was evaluated by decrease in muscle mass relative to individual contralateral level.

### Western blotting

Mice were anesthetized and muscles were quickly isolated, and 10 to 20 mg pieces of the muscle tissues were homogenized in a SDS-PAGE sample buffer containing 2% SDS and 1% **β**-mercaptoethanol, using a mechanical homogenizer (Pro Scientific, Oxford, CT, USA). Protein samples were heated at 85°C for five minutes and centrifuged to remove the insoluble materials. Protein samples were then resolved on either 10% or 15% SDS-PAGE with an acrylamide to bis-acrylamide ratio of 29:1. The resulting gels were transferred to nitrocellulose membranes and probed with primary antibodies as indicated. The signals of the protein bands were visualized using enhanced chemiluminescence method (GE Amersham, Pittsburgh, PA, USA). Relative quantity of protein was analyzed by densitometry using NIH Image J program (Bethesda, Maryland, USA). Primary antibodies used in this study were as follows: anti-MST1 (Cell Signaling Technology, Danvers, MA, USA), anti-MST2 (Abcam, Cambridge, UK), anti-GAPDH (Sigma, St. Louis, MO, USA), anti-LC3b (Cell Signaling Technology, Danvers, MA, USA), anti-Ser207-phosphorylated FOXO3a (Invitrogen, Grand Island, NY, USA). A monoclonal antibody against the C-terminal region of mouse FOXO3a was prepared and used to detect FOXO3a protein level from muscle lysates [51].

### Quantitative PCR

Total RNA from the freshly isolated muscle tissues were extracted using a Trizol method according to the manufacturer's instruction (Invitrogen). Two micrograms of RNA were converted to first strand cDNA using M-MLV reverse transcriptase (Promega, Madison, WI, USA) following the manufacturer's instruction. The quantitative PCR were carried out with a Corbett 6200 machine (Valencia, CA, USA) using a three-step protocol and PCR reaction for each sample was performed in triplicate. Primers used in quantitative PCR are as follows:

Mouse MST1:

Forward-GAACCATGAAAAGAAGAGATGAGA, Reverse-GTCCAGCTCTTGAGAAACTCAT;

Atrogin-1:

Forward-TGAATAATCCCAGCACACGA, Reverse-ATCGGCAACTGCATCTCTTC;

MuRF-1:

Forward-TAACTGCATCTCCATGCTGGTG, Reverse-TGGCGTAGAGGGTGTCAAACTT;

LC3:

Forward-CGGCTTCCTGTACATGGTTT, Reverse-ATGTGGGTGCCTACGTTCTC;

Bnip3:

Forward-CACCTTCTGATGAAGATTTGGA, Reverse-GGAACACCGCATTTACAGAA.

For each primer pair, analysis of the melting curve was performed to verify the specificity of the PCR product and reaction conditions. Cycle numbers were measured using Rotor-Gene 6000 software (Valencia, CA, USA) and results were analyzed using the 2^-ΔΔCt ^method.

### Immunofluorescence

Muscles were freshly isolated and mounted in a plastic bowl containing OCT (Thermo Scientific, Rockfield, IL, USA), and the orientation of the muscle fibers was maintained to ensure the sectioning is performed in a transverse manner. The mounted muscle tissues were frozen in isopentane pre-chilled at -159°C for 30 to 40 seconds and stored at -80°C. Transverse sectioning of the muscle tissues was performed using a Leica CM3050S cryostat (Wetzlar, Germany). Serial sections with 10 nm thickness were used for immunostaining and H&E staining following a protocol as described previously [52]. Briefly, after being rehydrated in PBS containing 0.05% Tween-20 (PBST) for 15 minutes and blocked in PBST containing 3% BSA for 30 minutes, muscle sections were probed with primary antibodies at 4°C overnight. After three washes with PBST for 10 minutes each, muscle sections were incubated with fluorescent chromophore conjugated secondary antibodies. After three washes with PBST, muscle sections were mounted and images were captured using a Leica SP5 confocal microscopy. Muscle fiber areas were quantified using NIH Image J software (Bethesda, Maryland, USA). Primary antibodies used in immunostaining were as followings: anti-MHC I (Developmental Hybridoma Bank, Iowa, USA) and anti-alpha-laminin (Cambridge, UK).

## Abbreviations

Gas: Gastrocnemius; KO: knockout; MST: Mammalian Sterile 20-like kinase; PBS: phosphate-buffered saline; PBST: phosphate-buffered saline containing 0.05% Tween-20; PTM: post-translational modifications; Sol: soleus; TA: tibial anterior

## Competing interests

The authors declare that they have no competing interests.

## Authors' contributions

BW, ZY and GJ designed the experiments. BW and WD performed the experiments. DL contributed when this project was initiated. BW, ZY and GJ analyzed the data and prepared the manuscript. ZY is also a member of the Chinese Hippo Consortium. All authors have read and approved the final manuscript.

## Supplementary Material

Additional file 1 **MST1 protein level is increased in unloading-induced muscle atrophy**. **A**. Representative blots show MST1, but not MST2, protein level was significantly increased in gastrocnemius muscles seven days after hindlimb unloading. Neither MST1 nor MST2 protein level was statistically altered in gastrocnemius muscles after 48-hour starvation. **B**. Quantification of MST1 and MST2 protein levels in hindlimb unloading- and starvation-induced muscle atrophy. *, *P *< 0.05 by two-tailed Student's test. Click here for file

Additional file 2 **Mass of fast-dominant TA and gastrocnemius muscles are decreased in adult MST1 KO mice**. Soleus, EDL, TA and Gas muscles were isolated from three-to five month-old WT (n = 6) and MST1 KO (n = 7) mice. Mass data of the muscles was normalized to body weight data of the mice. *, *P *< 0.05 compared with wild type by two-tailed Student's test. Click here for file

Additional file 3 **MST1 kinase is not involved in denervation-induced atrophy of slow dominant muscle. A **and **B**. Western blotting and densitometry showed neither MST1 nor MST2 kinase protein level was altered in denervated soleus muscles. Click here for file

Additional file 4 **MST1 deficiency does not affect type I fiber composition in diaphragm muscles**. MHC I staining of muscle cryosections showed similar type I fiber content in adult WT and MST1 KO diaphragm muscles. Click here for file
