# Supplementary figures and images for: MST1, a key player, in enhancing fast skeletal muscle atrophy
Source: BMC Biol. 2013 Feb 1;11:12. doi: 10.1186/1741-7007-11-12 (PMC3606410; doi:10.1186/1741-7007-11-12)

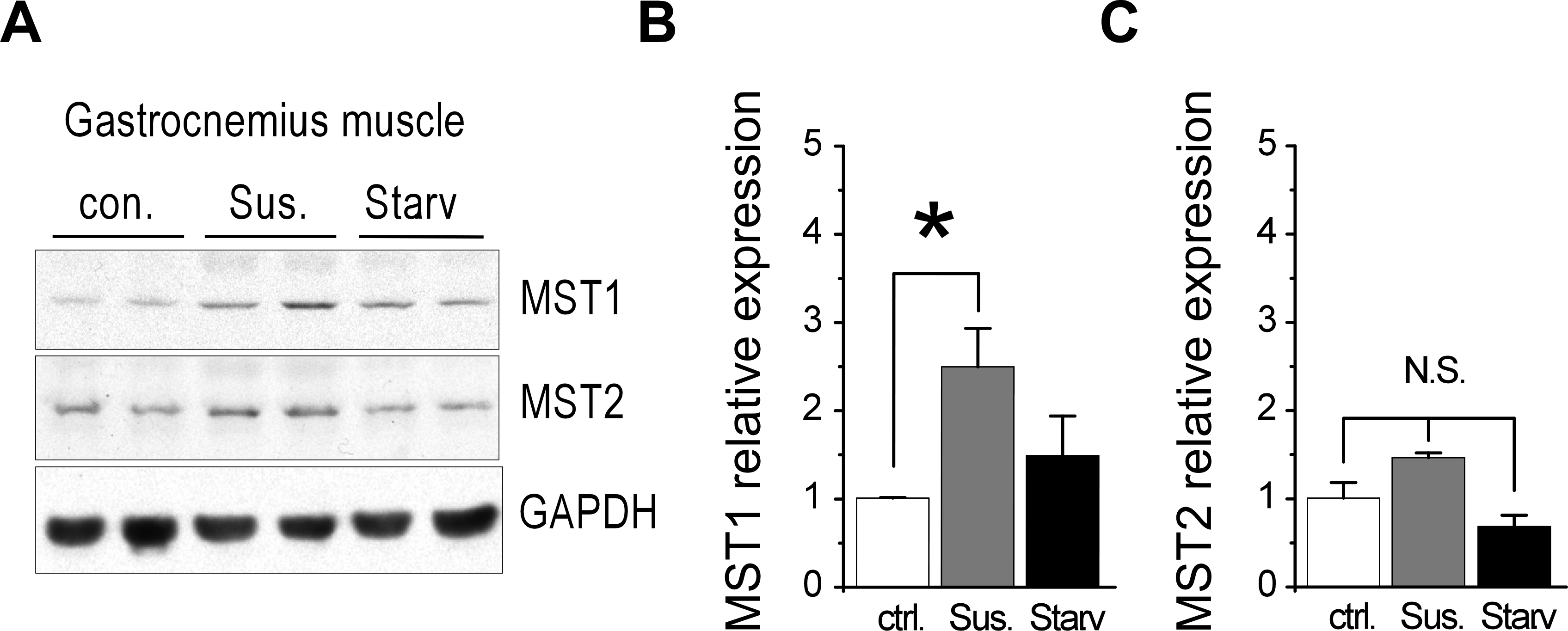

Supplement: Additional file 1 — MST1 protein level is increased in unloading-induced muscle atrophy. A. Representative blots show MST1, but not MST2, protein level was significantly increased in gastrocnemius muscles seven days after hindlimb unloading. Neither MST1 nor MST2 protein level was statistically altered in gastrocnemius muscles after 48-hour starvation. B. Quantification of MST1 and MST2 protein levels in hindlimb unloading- and starvation-induced muscle atrophy. *, P < 0.05 by two-tailed Student's test. [file 1741-7007-11-12-S1.JPEG]

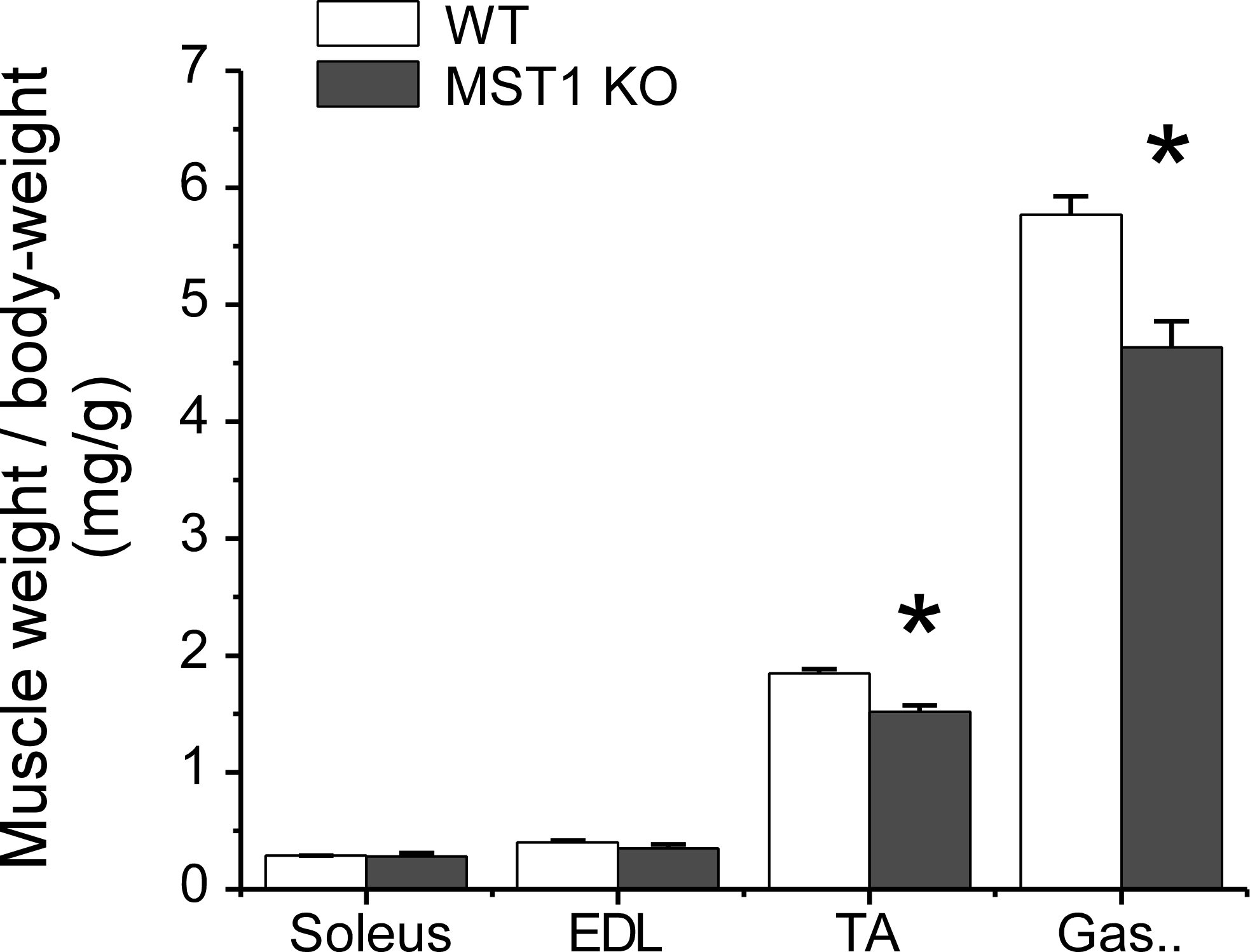

Supplement: Additional file 2 — Mass of fast-dominant TA and gastrocnemius muscles are decreased in adult MST1 KO mice. Soleus, EDL, TA and Gas muscles were isolated from three-to five month-old WT (n = 6) and MST1 KO (n = 7) mice. Mass data of the muscles was normalized to body weight data of the mice. *, P < 0.05 compared with wild type by two-tailed Student's test. [file 1741-7007-11-12-S2.JPEG]

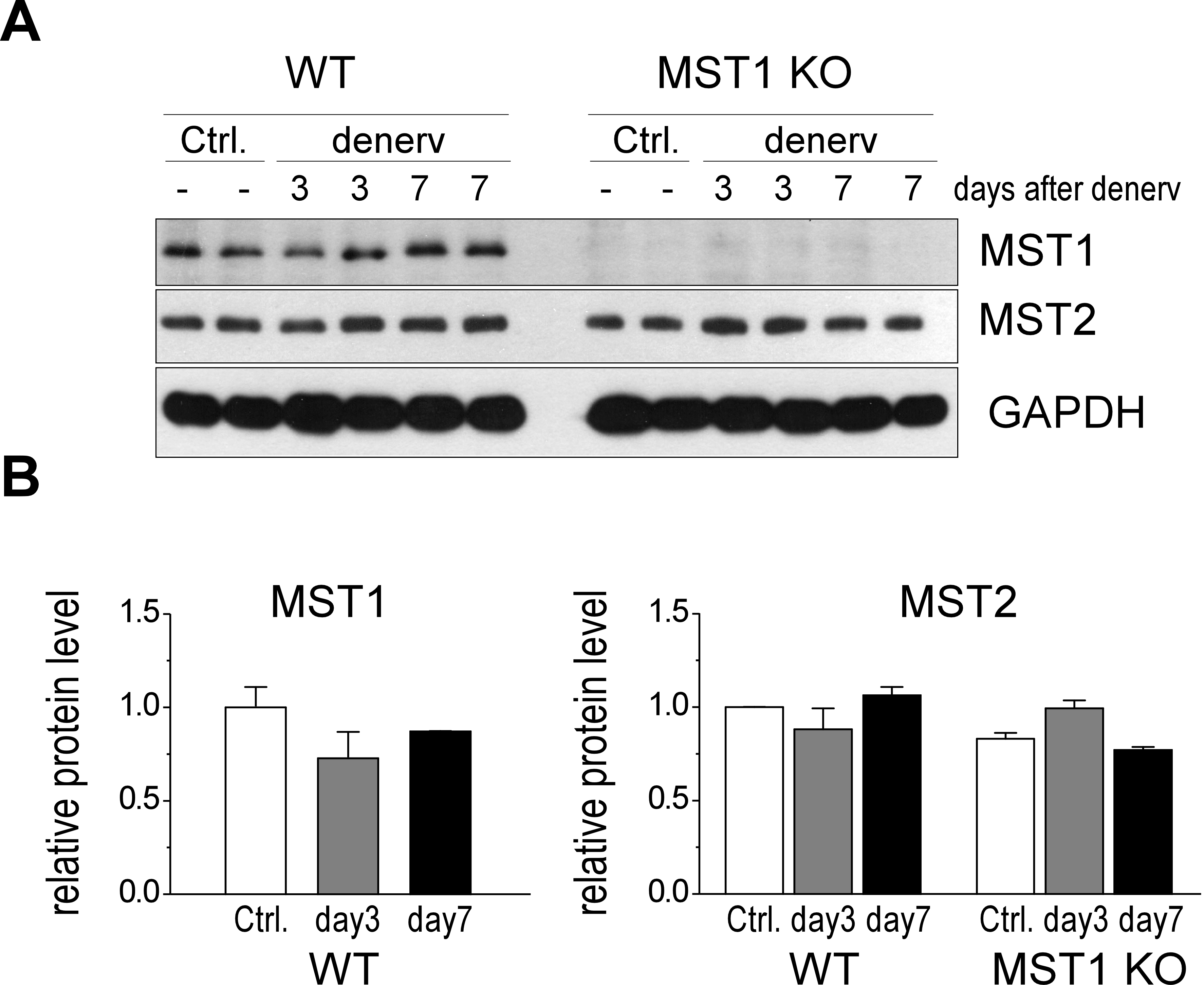

Supplement: Additional file 3 — MST1 kinase is not involved in denervation-induced atrophy of slow dominant muscle. A and B. Western blotting and densitometry showed neither MST1 nor MST2 kinase protein level was altered in denervated soleus muscles. [file 1741-7007-11-12-S3.JPEG]

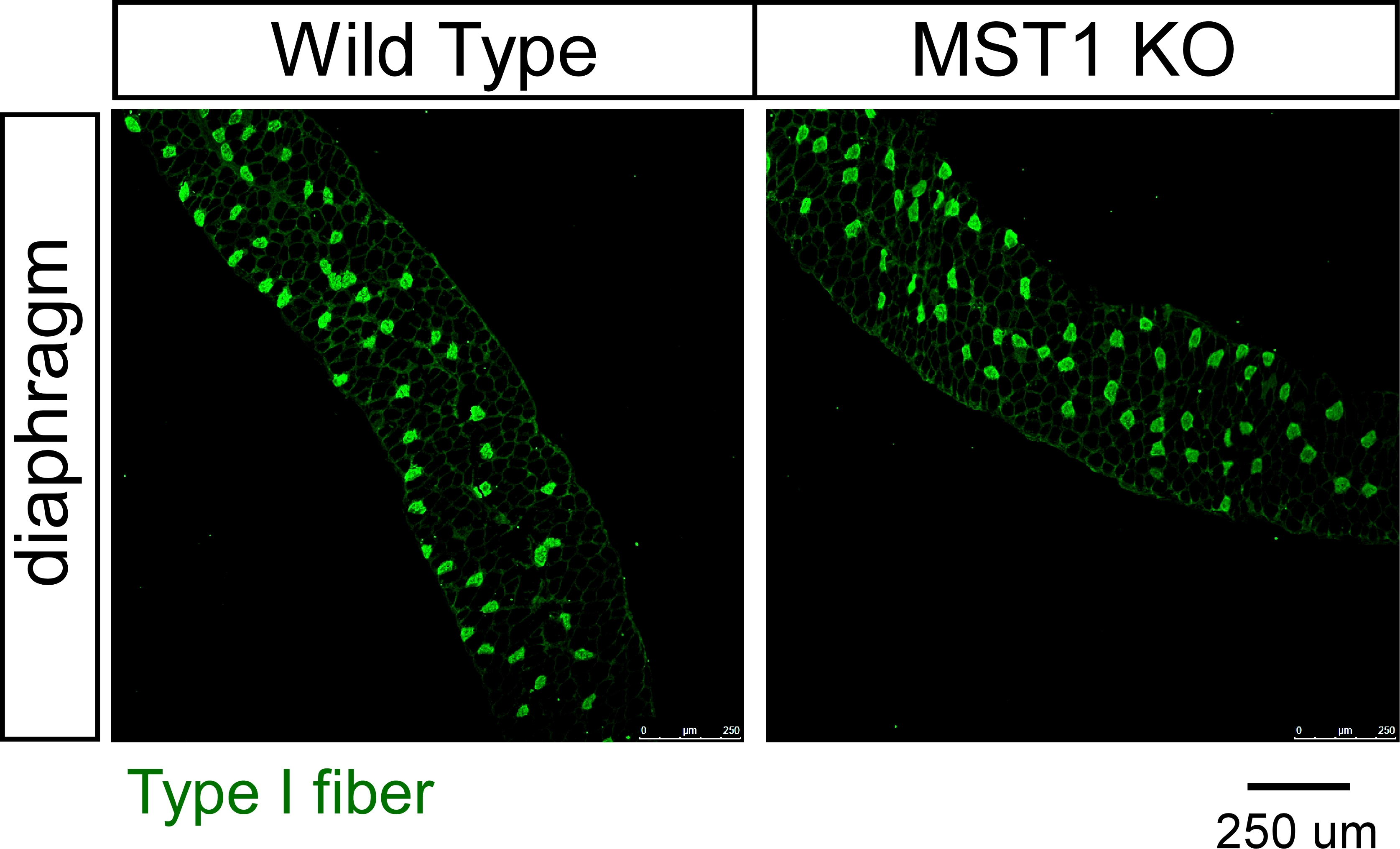

Supplement: Additional file 4 — MST1 deficiency does not affect type I fiber composition in diaphragm muscles. MHC I staining of muscle cryosections showed similar type I fiber content in adult WT and MST1 KO diaphragm muscles. [file 1741-7007-11-12-S4.JPEG]
